# Diphtheria outbreak with high mortality in northeastern Nigeria

**DOI:** 10.1017/S0950268813001696

**Published:** 2013-07-18

**Authors:** N. C. BESA, M. E. COLDIRON, A. BAKRI, A. RAJI, M. J. NSUAMI, C. ROUSSEAU, N. HURTADO, K. PORTEN

**Affiliations:** 1 Epicentre, Paris, France; 2 Médecins Sans Frontières, Abuja, Nigeria; 3 School of Medicine, Department of Medicine, Section of Infectious Diseases, Louisiana State University Health Sciences Center, New Orleans, LA, USA; 4 Médecins Sans Frontières, New York City, NY, USA; 5 Médecins Sans Frontières, Paris, France

**Keywords:** Community outbreaks, diphtheria, vaccine-preventable diseases

## Abstract

A diphtheria outbreak occurred from February to November 2011 in the village of Kimba and its surrounding settlements, in Borno State, northeastern Nigeria. We conducted a retrospective outbreak investigation in Kimba village and the surrounding settlements to better describe the extent and clinical characteristics of this outbreak. Ninety-eight cases met the criteria of the case definition of diphtheria, 63 (64·3%) of whom were children aged <10 years; 98% of cases had never been immunized against diphtheria. None of the 98 cases received diphtheria antitoxin, penicillin, or erythromycin during their illness. The overall case-fatality ratio was 21·4%, and was highest in children aged 0–4 years (42·9%). Low rates of immunization, delayed clinical recognition of diphtheria and absence of treatment with antitoxin and appropriate antibiotics contributed to this epidemic and its severity.

## INTRODUCTION

In August 2011, a cluster of deaths in children following an illness characterized by a swollen neck was reported at Biu General Hospital in Borno State, Nigeria. Most cases lived in the village of Kimba (population 1553), about 50 km south of the city of Biu. This cluster of deaths prompted an investigation at the hospital and in the community; pharyngeal swabs were collected from nine patients with classic pseudomembranes at the time of the initial investigation and tested at the Institut Pasteur in Paris. One sample grew *Corynebacterium diphtheriae* biovar *mitis*, positive for the *dtxR* and *tox* genes, confirming the clinical syndrome as diphtheria. In response to this cluster of cases, beginning in September 2011, the Nigerian Ministry of Health and Médecins Sans Frontières (MSF) offered case management. The absence of diphtheria antitoxin (DAT) at a national level and subsequent shipping and importation delays from France (the most readily available source) meant that DAT was never available during the outbreak. A mass vaccination campaign was held in Kimba in November 2011, with a follow-up campaign in March 2012.

Diphtheria is caused by the Gram-positive rod *C. diphtheriae*, and is characterized by laryngitis, pharyngitis or tonsillitis, in the presence of an adherent membrane of the tonsils, pharynx and/or nose [1]. Up to 25% of cases develop myocarditis, and the disease can also affect the peripheral nervous system, sometimes leading to temporary paralysis [2]. The pathogenicity of *C. diphtheriae* is due to an extracellular toxin, and individuals with incomplete immunization or low antitoxin antibody levels are most susceptible to infection [3]. Treatment of diphtheria consists of antitoxin and either penicillin or erythromycin, although Nigeria does not currently regularly stock DAT [4].

Diphtheria control is primarily based on prevention of infection by ensuring high population immunity through immunization. The occurrence of diphtheria outbreaks reflects inadequate coverage of national childhood immunization programmes [3]. The most recent major outbreak of diphtheria, with over 150 000 cases, was seen in the 1990s in the countries of the former Soviet Union, at a time when vaccination coverage was declining during the setting of political change [5]. Although diphtheria is declining or has been eliminated from many developed countries following widespread immunization programmes, it remains endemic in many developing countries [6–8]. This is in large part due to inadequate vaccine coverage, which was estimated at 71% in the World Health Organization's (WHO) African region and 75% in the South East Asian region [9]. India is a major focus, reporting 71% of the 4880 cases reported in the world in 2011 [10]. Several recent outbreaks have been described there, usually consisting of fewer than 100 cases, with case-fatality ratios (CFRs) ranging from 3% to 31%, and generally occurring in the setting of low (50–70%) vaccine coverage [11–13].

The WHO recommends a series of three doses of diphtheria, tetanus toxoids, and pertussis (DTP) vaccine beginning at age 6 weeks. Additional booster doses in childhood extend the duration of immunological protection [14]. In Nigeria, the Expanded Programme on Immunization (EPI) recommends doses of DTP vaccine at ages 6, 10 and 14 weeks [15]. In 2011, national coverage rates for the first and third doses of DTP in Nigeria were estimated at 53% and 47%, respectively [9, 16].

Kimba village and its surroundings are home to a semi-nomadic population which is absent for long periods, but which regularly returns to the same homes and villages. Access to healthcare is limited, and local vaccine coverage rates for diphtheria were <1% in 2011. In 2006, Nigeria reported 312 cases of diphtheria [10]. Since then, sporadic cases in several different localities have been described in the literature, but have not been declared in regular country-wide reporting to the WHO [3, 17].

Here we report the results of a retrospective household survey undertaken in Kimba village and its surroundings to better describe the extent and clinical characteristics of this outbreak.

## METHODS

### Setting

The survey was conducted in the village of Kimba, a municipality of Biu Local Government Area, and seven surrounding settlements located within a 5 km radius of the village of Kimba. Different case-finding methodologies were used in Kimba and its surrounding settlements.

### Household survey

An exhaustive, retrospective household survey was conducted in Kimba village during 9–12 December 2011. One adult in each household was interviewed using a standardized questionnaire. The initial screening consisted of asking the respondent whether, at any time between February 2011 and the day of the survey, any household member had developed any of the following signs or symptoms: fever, sore throat, swollen neck, difficulty breathing, drooling saliva and/or whitish membrane of the tonsil. For each individual with a positive initial screening, we collected additional information, including demographic data, detailed symptoms, time-course of the illness, type of treatment received, laboratory results, immunization status and patient outcomes.

In the seven settlements surrounding Kimba village, the survey took place on 13–14 December 2011. In these settlements, the survey was not exhaustive. Instead, village elders were asked to identify all households where members had suffered from the above symptoms. Interviewers then went to each household identified by village elders and continued the same process described above, using the same standardized questionnaire.

### Definitions

A probable case of diphtheria was defined as any person residing in the village of Kimba and its surrounding settlements between February and December 2011 who developed:

• a sore throat with difficulty in swallowing, difficulty in breathing, or drooling saliva (laryngitis or pharyngitis or tonsillitis);
• and a whitish or greyish layer on the tongue, palate, throat or nose (adherent membrane of the tonsils, pharynx and/or nose);
• and at least one of the following: swelling or oedema of the neck (bull neck), stridor, submucosal or skin petechial haemorrhage and/or motor paralysis 1–6 weeks after onset of symptoms, and any death associated with the symptoms mentioned above.

A confirmed diphtheria case was a probable case confirmed by laboratory isolation of toxigenic *C. diphtheriae*. A probable diphtheria death was defined as a probable diphtheria case as defined above that died within 6 weeks of onset of symptoms, and a confirmed diphtheria death was defined as a confirmed diphtheria case as defined above that died within 6 weeks of onset of symptoms.

### Data collection and data analysis

Data were collected by locally recruited health workers fluent in English and Hausa who had received training on the survey protocol. Interviews were conducted in Hausa. Data were entered using Microsoft Excel (Microsoft, USA) and analysed using SAS (SAS Institute Inc., USA).

## RESULTS

Each of the 140 households of Kimba village was interviewed; the total population was 1553 persons. In the surrounding settlements, village elders identified 38 households in which at least one person had suffered the symptoms listed in the case definitions. One respondent in each of these 38 households was also interviewed. Hereafter, ‘Kimba’ refers to the village of Kimba and its surrounding settlements, unless otherwise specified.

Initial screening at the households identified 220 individuals suffering from symptoms suspicious for diphtheria during the recall period. The full study questionnaire was administered to each of these 220 individuals, or their parents/guardians. Of these, 97 were classified as probable cases and one was classified as a confirmed case. The remaining 122 individuals did not fully meet the criteria set out in the case definitions.

**Fig. 1.**
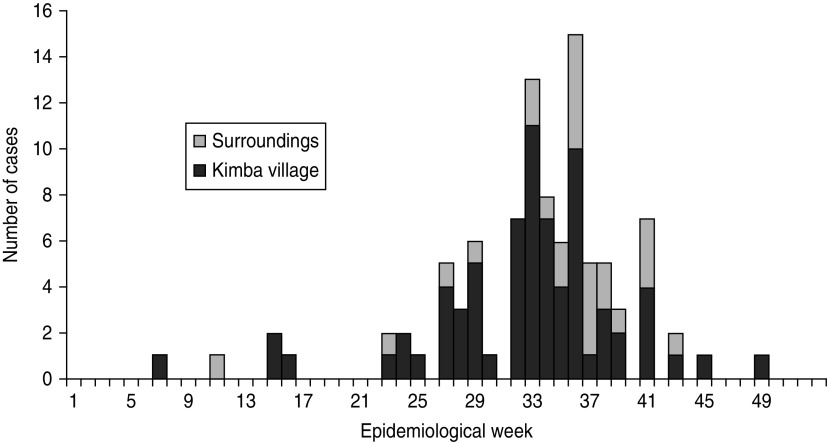
Probable and confirmed diphtheria cases by geographical origin and epidemiological week, Kimba, Borno State, Nigeria, February–December, 2011.

The first probable diphtheria case occurred in February 2011. Cases continued until November 2011, with the highest weekly incidence occurring between weeks 32 and 36 (Fig. 1). Seventy-three (74·5%) of the cases were identified in Kimba village proper, corresponding to an attack rate of 4·7% over a 10-month period in the village. Characteristics of cases are presented in Table 1. Only 4/98 cases (4·1%) reported ever having received even a single dose of diphtheria vaccine prior to February 2011. The majority (51·0%) of all cases reported being treated exclusively with traditional products, either by self-medication or in consultation with a traditional healer.

**Table 1.** Description of diphtheria cases (N = 98), Kimba, Nigeria, 2011

	*n*	%
Sex		
Male	41	41·8
Female	57	58·2
Age (years)		
0–4	21	21·4
5–9	42	42·9
10–14	16	16·3
⩾15	19	19·4
Treatment*		
Antibiotic only†	27	27·6
Traditional treatment only	50	51·0
Both antibiotic† and traditional	14	14·3
Unknown	7	7·1
Outcome		
Survived	75	76·5
Death	21	21·4
Unknown	2	2·1

* No patient was reported having been treated with diphtheria antitoxin, penicillin, or erythromycin.

† All reported the antibiotic to have been amoxicillin and/or ceftriaxone.

A total of 21 deaths were reported among the 98 confirmed and probable cases, a CFR of 21·4% (Table 2). Twelve deaths (CFR 16·4%) were reported in Kimba village proper, and nine deaths (CFR 36·0%) were reported in the surrounding settlements. The CFR was higher in males (31·7%) than in females (14·0%). The highest CFR (42·9%) was seen in children aged < 5 years; CFR decreased with increasing age, and no deaths were reported in cases aged ⩾15 years.

**Table 2.** Diphtheria case-fatality ratios by selected characteristics, Kimba, Nigeria, 2011

	Cases	Deaths	Case-fatality ratio
Location			
Kimba village	73	12	16·4%
Surroundings	25	9	36·0%
Sex			
Male	41	13	31·7%
Female	57	8	14·0%
Age (years)			
0–4	21	9	42·9%
5–9	42	10	23·8%
10–14	16	2	12·5%
⩾15	19	0	0%
Care			
No formal care	84	15	17·9%
Outpatient	7	4	57·1%
Inpatient	7	2	28·6%
Total	98	21	21·4%

Of the 91 patients who reportedly received any form of specified treatment (traditional or allopathic), the mean length of time between the onset of symptoms and the beginning of treatment was 2·8 days. A total of 14 cases were brought to and treated at Biu General Hospital, seven as outpatients and seven as inpatients. For cases treated at the hospital, the average length of time between onset of symptoms and beginning of treatment was 3·1 days. None of the cases treated at the hospital received a diagnosis of diphtheria; each received a working diagnosis of mumps or parotitis. Their antibiotic treatment consisted of amoxicillin and/or ceftriaxone; none received DAT, erythromycin, or penicillin.

An additional 30 individuals with an initial positive screen during the survey met the first and third criteria set out in the case definitions, i.e. sore throat plus at least one additional symptom, but no adherent membrane was reported. The development of signs and symptoms in these 30 individuals followed the same temporal trends (90% occurred between weeks 26 and 41) as those observed in the 98 diphtheria cases (86% occurred between weeks 26 and 41). Nonetheless, these 30 individuals were not included as cases in the present analyses because the absence of adherent membranes did not meet our case definitions. Only one death was reported in these 30 individuals.

## DISCUSSION

This outbreak was probably the consequence of very low childhood vaccination rates, as well as lack of availability of booster vaccinations for older children and adults. Kimba, like many other remote areas in Nigeria, is not well-covered by the EPI, with routine coverage for DTP reported as <1% in 2011.

Our results illustrate the long time period between the first cases of diphtheria (February 2011) and the eventual recognition of the clinical syndrome as being diphtheria (September 2011). As an uncommon disease, clinicians in the area were probably unfamiliar with its presentation. This led to subsequent delays in responses, including case management and vaccination activities, which did not happen until November 2011, over 9 months after the first case was seen in the village. Some of this delay is also probably explained by the lack of access to healthcare in Kimba village, as the first cases to seek care in Biu General Hospital, 50 km away, did so in August 2011, several months after the first cases were seen in Kimba.

The CFR during this epidemic was elevated, probably exacerbated by the complete absence of effective diphtheria treatments. None of the cases identified in this survey received antitoxin or the recommended antibiotics. The first and only laboratory confirmation of diphtheria came in September. Most probable diphtheria deaths occurred in the village before the patients could be brought to the hospital, and most inpatient fatalities occurred within 72 h of admission. This underscores the short interval for therapeutic intervention, and emphasizes the importance of improving access to care in an epidemic setting. This zone has a highly seasonal cycle of malnutrition, of which the traditional peak coincided with this outbreak. No formal nutritional evaluations were conducted in Kimba in 2011, although the season was not reported to be abnormally severe. While it is possible that seasonal malnutrition may have influenced the mortality in this outbreak, this retrospective investigation does not allow us to comment on any potential links.

The high CFR reported in this study may also be partly due to an overly specific case definition of diphtheria cases. The presence of a membrane is an important clinical feature of diphtheria; however, it is more reliable if observed by a clinician. In Kimba, it is possible that parents did not look in their children's throats and/or could not see membranes. If this were the case, our retrospective survey may not have captured all diphtheria cases, and our observed CFR may be higher than it actually was during the outbreak. This hypothesis is supported by the temporal concurrence of the 98 confirmed and probable cases with the 30 individuals who had symptoms suggestive of diphtheria but who were ultimately discarded because of lack of visible adherent membranes. Our experience with the highly specific case definition, i.e. presence of membrane in an already confirmed epidemic, would suggest using a less specific case definition in similar settings.

Other limitations of this study include the possibility that some respondents may not have had complete knowledge of the health status of the household member(s) for whom they were reporting signs and symptoms. It is also possible that recall bias could have affected the reporting of events that, in most instances, occurred several months previously. Furthermore, difficulties in translating medical terms into Hausa may have possibly introduced some misclassification bias, especially between the 98 probable and confirmed cases and the 30 discarded cases. However, our investigation leaves little doubt as to the occurrence of the outbreak.

This diphtheria outbreak in northeastern Nigeria illustrates several prevailing challenges in providing care in isolated rural areas in many developing countries. These challenges include, but are not limited to, poor vaccination coverage, weak preventable disease surveillance systems, clinicians' unfamiliarity with uncommon diseases, and limited access to healthcare facilities.
